# Application of transverse acetabular ligament in total hip arthroplasty: a systematic review

**DOI:** 10.1186/s12891-023-06410-0

**Published:** 2023-04-13

**Authors:** Dongfang Ning, Feng Xu, Zhongxing Zhang, Xiaolong Yang, Jun Wei

**Affiliations:** Department of Bone and Joint Surgery, Liuzhou Municipal Liutie Central Hospital, Liuzhou, Guangxi 545007 China

**Keywords:** Transverse acetabular ligament (TAL), Total hip arthroplasty (THA), Safe zone, Acetabular component orientation

## Abstract

**Introduction:**

In total hip arthroplasty (THA), the correct position of the acetabular component directly determines the outcome of the surgery, or the success of the surgery. Therefore, how to accurately locate the position of the acetabular component has become a very critical step in THA. As an important anatomical structure of the hip joint, the transverse acetabular ligament (TAL) is helpful for acetabular component orientation in THA. The aim of this systematic review was to investigate application of TAL in THA.

**Materials and methods:**

A systematic literature search of PUBMED, EMBASE, and Cochrane Library was performed (January and February 2023) using keywords “total hip arthroplasty,” “total hip replacement,” “total hip replacements,” “total hip arthroplasties,” “total hip prosthesis,” and “transverse acetabular ligament” in all possible combinations. Reference lists of included articles were reviewed. Study design, surgical approach, patient demographics, TAL identification rate, appearance of the TAL, anteversion and inclination angle and rate of dislocations were recorded.

**Results:**

In total, 19 studies met the screening criteria. Study designs were prospective cohorts (42%), retrospective cohorts (32%), Case series (21%), and randomized controlled trial (5%). Twelve of the 19 (63.2%) studies investigated the application of TAL as an anatomical landmark for locating acetabular component position in THA. Analysis revealed that TAL is a reliable anatomical landmark for acetabular component orientation within the safe zone in THA.

**Conclusions:**

TAL can reliably be used to align the acetabular component in the safe zone for anteversion and inclination in THA. However, TAL has individual variation influenced by some risk factors. More randomized controlled studies with larger numbers of patients are needed to investigate the precision and accuracy of TAL as an intraoperative landmark in THA.

**Level of evidence:**

IV.

## Introduction

In THA, accurate orientation of the acetabular component can reduce dislocation and impingement, achieve stability, maximize range of motion, improve survival [1–5]. So accurate orientation of acetabular component plays a very important role in determining the outcome of THA surgery. Lewinnek et al. defined the “safe zone” for acetabular component placement as 5°-25°anteversion angle and 30°-50°inclination angle. When acetabular components were placed within the safe zone, the dislocation rate decreased significantly [6]. Although this safe zone is a reliable reference, there is no clear definition in the anatomy and biomechanics of the hip joint. The orientation of the pelvis is not absolutely fixed and may change due to intraoperative factors, such as the positioning of the operating table, dislocation of the hip joint, patient’s position, and incision traction exposure, etc. [7]. Whether done by freehand or with mechanical guides, ignoring these factors and simply referring to the safe zone can lead to misjudgment of acetabular component orientation. With the development of the Times, 3D printing technology, computer navigation equipment and orthopedic robot have been applied in THA, which further improves the surgical accuracy and tends to be minimally invasive. However, the high technical level and high price are not conducive to the development of primary hospitals and increase the burden of hospitalization costs of patients [8]. Some authors have recommended that the TAL can be used as anatomic reference landmark for acetabular component orientation [9–12]. By this method, they could reduce the dislocation rate and obtain good orientation of the acetabular component [13, 14]. However, some scholars were skeptical about using TAL as a reference landmark for optimal acetabular component orientation [15–17]. They thought that TAL’s reliability as a landmark for acetabular component orientation needed more research to confirm. This systematic review aims to investigate the existing literature on applications of TAL in THA.

## Materials and methods

### Search criteria

The electronic database PubMed/MEDLINE, EMBASE, and Cochrane were comprehensively searched for publications from January 1980 to February 2023 utilizing keywords pertinent to total hip arthroplasty (THA) and transverse acetabular ligament (TAL). Only abstracts that evaluated the value of TAL as a guide for acetabular component orientation in THA were included in this analysis.

### Inclusion and exclusion criteria

The inclusion criteria were: (1) Full text available, (2) studies written in English, (3) studies describing human subjects of any age and gender, (4) studies investigating the value of TAL as a guide for acetabular component orientation in THA.

**Fig. 1:**
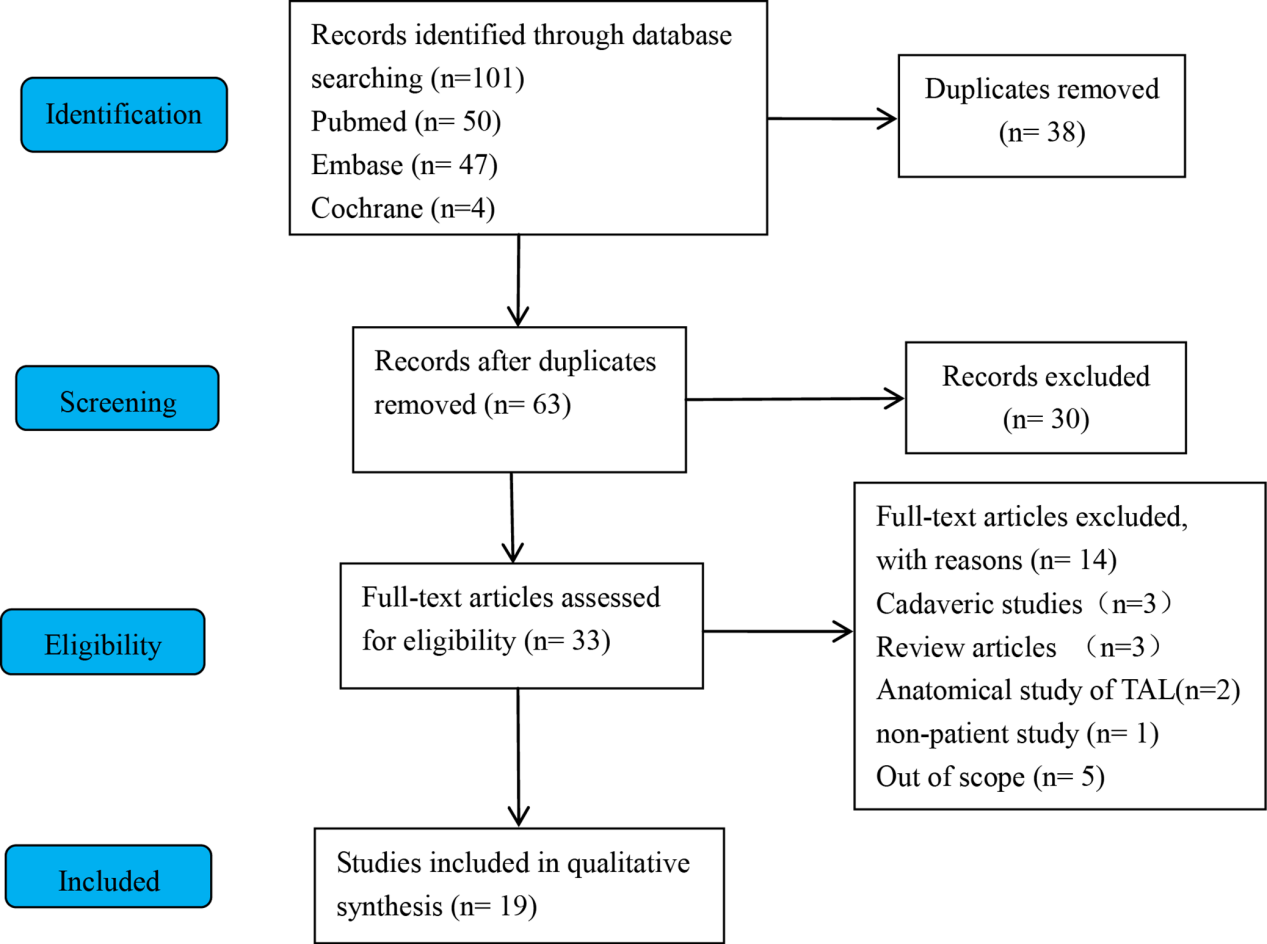
Systematic review flow diagram

The exclusion criteria were:(1) case report studies, expert opinions or letter to the editor, (2) non-English language articles, (3) animal studies, (4) non-patient study (e.g. biometric computational simulation(s)), (5) nonindexed and unpublished data, (6) studies of TAL not as a guide for acetabular component orientation in THA, (7) cadaveric studies, (8) review articles or meta-analysis, (9) non-full-text articles. The process of literature search and selection of articles for this review are provided in Fig. 1.

### Data collection

Initial review of the data was performed by two independent reviewers, the following information was collected for each study: author, year published, journal, study design, number of patients, number of hips, gender, mean age, operative approach, patient position, appearance of the TAL, anteversion and inclination angle of the acetabular component, mean final follow-up time, rate of dislocations. All authors performed relevant paper selection that met inclusion and exclusion criteria. Discrepancies between the authors were resolved by discussion.

### Quality assessment

The level of evidence for included studies was assessed by us the Oxford Center for Evidence-based Medicine Levels of Evidence [18]. The methodological quality of included studies and the different types of detected bias were determined by independent reviewers using modified Coleman methodology score [19].

The paper has been reported which is consistent with PRISMA (Preferred Reporting Items for Systematic Reviews and Meta-Analysis) and AMSTAR (Assessing the methodological quality of systematic reviews) Guidelines.

## Results

### Search process and results

Using the search criteria listed, 101 studies were identified (Fig. 1). Among these, 38 duplicate studies were identified and removed from them. Following the removal of duplicate studies, there are still 63 studies applying the predetermined inclusion and exclusion criteria. Following application of these criteria, 33 articles were performed a full text screening process, with 14 articles excluded from final analysis. Finally, 19 Studies were included in qualitative synthesis for further analysis [9, 12, 13, 17, 20–34]. The details regarding these studies, are provided in Table 1.

**Table 1:** Demographics and study description

Author(year)	Journal	Study design	Patients/ hips N	Gender(M/F)	Mean age (Y)	Approach	position	TAL visible	Anteversion angle	Inclination angle	FU (M)	Dislocations (%)	Level of evidence
Meermans G et al. (2014) [20]	Bone Joint J	RCT	80/80	34/46	69.6	posterior	lateral decubitus	100%	17(5–25)	45(30–63)	24	0	I
Salal MH (2017) [21]	J Coll Physicians Surg Pak	Case series	31/31	22/9	64 ± 5.28	posterolateral	lateral decubitus	100%	14.7(5.7–24.4)	NA	1	0	IV
Hideaki Miyoshi et al. (2012) [22]	The Journal of Arthroplasty	Retrospective study	46/47	8/38	67.7	posterolateral	lateral decubitus	81.6%	21.2 ± 4.1(10.5–28.8)	38 ± 7 (22.1–54.1)	17	0	III
Yoon BH et al. (2016) [23]	Journal of Orthopaedic Science	Retrospective study	81/90	33/48	33.3 ± 9.8	NA	NA	NA	11.8 ± 4.5(0-22.2)	NA	NA	NA	III
Abe H et al. (2012) [24]	Acta Orthopaedica	Prospective study	160/ 160	76/84	NA	NA	NA	100%	23(22–25)	66(64–67)	NA	NA	II
Fujita K et al. (2014) [25]	Bone Joint J	Prospective study	121/ 134	26/95	60.2	posterolateral	lateral decubitus	83.6%	22.7 ± 8.5(7.7–54.7)	NA	NA	NA	IV
LI L et al. (2021) [26]	zhongguo Zuzhi Gongcheng Yanjiu	Retrospective study	192/ 384	84/108	54.52	NA	NA	NA	12.9 ± 3.3	NA	NA	NA	III
A.R. Griffin et al. (2014) [27]	The Journal of Arthroplasty	Retrospective study	160/ 218	79/81	58.7	NA	N	NA	20.5° ± 7.0°	NA	NA	NA	III
Archbold HA et al. (2006) [13]	Journal of Bone and Joint Surgery	Case series	1000/ 1000	463/537	68.3	posterolateral	lateral decubitus	99.7%	NA	NA	8–41	0.6	IV
Kumar V et al. (2014) [28]	Bone Joint J	Case series	512/ 512	282/230	67	posterior	lateral decubitus	100%	18.2 (1.0-36.7)	45.02(18.6–73)	33.6 (6-79.2)	0.78	IV
Ho KW et al. (2012) [29]	Arch Orthop Trauma Surg	Retrospective study	404/ 421	234/170	70 ± 9.8	posterior	lateral decubitus	100%	NA	45 ± 5.5	18–96	0.5	III
Ling T et al. (2021) [30]	BMC Musculoskeletal Disorders	Retrospective study	132/ 144	70/62	56.7	posterior	lateral decubitus	100%	21.7 ± 5.6 (6–37)	38.4 ± 6.9(20–56)	12.8 (3–36)	4.16	III
Idrissi ME et al. (2016) [12]	Acta Ortop Bras	Prospective study	21/21	NA	NA	Minopenposterior	lateral decubitus	100%	16.9 ± 5	39.85 ± 6	NA	0	IV
Epstein NJ et al. (2011) [17]	Clin Orthop Relat Res	Prospective study	63/64	59/4	65	posterolateral	lateral decubitus	47%	23.6 ± 9.9 (10–39)	41 ± 6.6 (28–54)	> 3	0	II
Archbold HAP et al. (2009) [31]	Current Orthopaedic Practice	Prospective study	40/40	NA	NA	posterolateral	lateral decubitus	100%	19.7 ± 8.1	48.0 ± 7.1	12–18	NA	IV
Deep K et al. (2021) [32]	Arthroplasty Today	Prospective study	99/99	41/58	67.6	posterior	lateral decubitus	100%	14.90 ± 8.22 (4–34)	42.60 ± 6.19 (24–56)	NA	NA	IV
Kalteis T et al. (2011) [9]	J Bone Joint Surg [Br]	Prospective study	39/39	15/24	67	DAA	lateral decubitus	100%	18(-1-36)	41(32–51)	NA	NA	IV
Agarwal A et al. (2020) [33]	Journal of Clinical Orthopaedics and Trauma	Prospective study	35/35	18/17	51.52	anterolateral	lateral decubitus	100%	23.8 ± 4.9 (13.9–29.7)	44.8 ± 4.9 (36.6–54.2)	12.7	0	II
Molho DA et al. (2022) [34]	Journal of the American Academy of Orthopaedic Surgeons	Case series	31/31	NA	NA	DAA	supine	100%	12.9 (6–22)	48.1 (38–62)	> 1.5	0	IV

N number, M male, F female, Y year, TAL transverse acetabular ligament, M month, FU follow-up, DAA direct anterior approach, RCT randomized controlled trial, NA not applicable

### Study design

There were 3 level II studies [17, 24, 33], 6 level III studies (retrospective cohorts) [22, 23, 26, 27, 29]–30], 1 level I RCT [20], and 9 level IV studies [9, 12–[13, 21, 25, 28, 31]–32, 34]. Of the level II papers are prospective study, one compared the anatomical anteversion of TAL and the TAL-guided acetabular component orientation in relation to disease and gender using 3D reconstruction of computed tomography(CT) images [24], one was a single comparison of the TAL vs. free-hand technique for acetabular component orientation [17], and the final study compared the TAL to mechanical angle guide device placing acetabular component [33]. The only level I RCT that compared freehand introduction of acetabular component with TAL-guided introduction for anteversion of acetabular component [20].

The mean modified Coleman methodology score for included studies was 53.4, which ranged from 33 [26, 27] to 73[28]. This result indicates that the overall methodological quality is low to medium level.

### Patient demographics

Among the 19 included studies, there were a total of 3,247 patients and 3,550 hips included. Sixteen studies (N = 3,155) reported patients’ gender distribution, of whom 45.0% were females, the average age across the studies was 64.8.

### Surgical approach

Fifteen of the 19 studies reported the surgical approach utilized during the procedure [9, 12, 13, 17, 20–22, 25, 28–34]. The posterior approach was the most utilized (86.6%), 2 studies [9, 34] are the DAA (13.3%) and the remaining 1 study is anterolateral approach(6.7%) respectively. There is only one study of the supine surgical position [34]. All patients in the rest studies were positioned in the lateral decubitus position with the pelvis stabilized with a lumbar and pubic support in the operating room. Their findings and conclusions all affirm the effectiveness of TAL in improving the acetabular component position. No clinical studies were found to compare and analyze the effect of TAL on improving the position of the acetabular component between DAA and posterior approach.

### TAL identification rate

Twelve of the 19 (63.2%) studies reported TAL identification rates in the literature, and all of them agreed on the application of TAL [9, 17, 21–26, 30, 32–34]; The details regarding these studies, are provided in Table 2. When TAL was used as an intraoperative landmark during THA there were a total of 1144 hips included to identify acetabular component anteversion alignment, and 343 hips included to identify acetabular component abduction alignment.

Excluding the literature with unclear preoperative diagnosis and etiological classification, 2247 hips (91.5%) included in various studies [9, 12–[13, 20–22, 24, 25, 28]–29, 31] were of inflammatory etiology, 208 cases (9.5%) were non- inflammatory hip joint. Only one literature [24] had a comparison of TAL localisation and orientation in inflammatory (OA)vs non-inflammatory hips(ON). In both groups, the TAL can be visualized intra-operatively. However, in non- inflammatory hip, TAL is a better intraoperative landmark for acetabular localization in THA than in inflammatory hip.

Of the measured value of anteversion angle of TAL, 79.1% (905 of 1144 hips) were within the safe zone (15 ± 10) as defined by Lewinnek. Of the measurements of the angle of acetabular component abduction, 81.3% (279 of 343 hips) were within the safe zone (40 ± 10) as defined by Lewinnek.

### Appearance of the TAL

Six of the 19 (31.6%) studies reported appearance of the TAL in the literature [12, 13, 17, 20, 22, 25], there were a total of 1306 hips included, of which 94.8% (N = 1238), the TAL can be visualized intraoperatively. In 3 studies [13, 20, 25], the appearance of the TAL was classified into four grades according to the scale by Archbold et al. [13]. Among the 3 studies that did (N = 1,174), 49.1% (N = 576) were Grade 1, TAL immediately visible; 31.7%(N = 372) were Grade 2, TAL covered by soft tissue; 17.1% (N = 201) were Grade 3, TAL covered by osteophytes; 2.1% (N = 25) were Grade 4, TAL not identified, even after adequate clearance.

**Table 2:** TAL identification rate reported in the literature

Author(year)	Study population type	Patients N	Hips N	TAL Identification Rate (%)	Reference standard	Measurement methods
Salal MH (2017) [21]	Patients	31	31	100 (anteversion)	Lewinnek’s safe zone	Radiographic measurement (CT)
Hideaki Miyoshi et al. (2012) [22]	Patients	46	47	100 (anteversion)	Lewinnek’s safe zone	Radiographic measurement (CT)
Yoon BH et al. (2016) [23]	Patients	81	90	91.2 (anteversion)	Lewinnek’s safe zone	Radiographic measurement (CT)
Abe H et al. (2012) [24]	Patients	80	80	OA 61 (anteversion) ON 91 (anteversion)	Lewinnek’s safe zone	The CT images and 3D template software
Fujita K et al. (2014) [25]	Patients	121	134	94.6 (anteversion)	Lewinnek’s safe zone	CT-based and surface registration-type navigation system
LI L et al. (2021) [26]	Patients	194	384	96 (anteversion)	Lewinnek’s safe zone	CT-based and surface registration-type navigation system
Ling T et al. (2021) [30]	Patients	132	144	73.6 (anteversion) 84 (inclination)	Lewinnek’s safe zone	Radiographic measurement
Epstein NJ et al. (2011) [17]	Patients	63	64	59 (anteversion) 83 (inclination)	Lewinnek’s safe zone	Radiographic measurement
Deep K et al. (2021) [32]	Patients	99	99	71.71(anteversion,inclination)	Lewinnek’s safe zone	Imageless computer-assisted navigation
Kalteis T et al. (2011) [9]	Patients	39	39	87(anteversion,inclination)	Lewinnek’s safe zone	An imageless navigation system
Agarwal A et al. (2020) [33]	Patients	35	35	100 (anteversion)	Lewinnek’s safe zone	Radiographic measurement (CT)
Molho DA et al. (2022) [34]	Patients	31	31	100 (anteversion) 90.4 (inclination)	Lewinnek’s safe zone	Radiographic measurement

N number, TAL transverse acetabular ligament, OA osteoarthritis, ON osteonecrosis, CT computerized tomography, 3D three dimensional

### Anteversion and inclination angle

Four of the 19 (21.1%) studies reported TAL anteversion in the literature [23, 25–27]. Three studies [25–27] found that the TAL correctly represented the main orientation of the acetabulum and was a useful landmark for acetabular component implanted within the safe zone. Therefore, TAL can be used as an anatomical landmark for locating acetabular component anteversion in THA. However, the remaining 1 study [23] reported that TAL anteversion has a large individual variation, and in a significant proportion of hips, the TAL anteversion are outside the safe zone of acetabular component. They were skeptical about using TAL as a reference mark for optimal acetabular component orientation.

Three of the 19 (15.8%) studies were controlled trial of two groups [17, 20, 33]. Two studies [17, 20] compared freehand introduction of acetabular component with TAL-guided introduction for anteversion of acetabular component. Meermans et al. thought the TAL may be used to obtain the appropriate acetabular component anteversion but not acetabular component inclination in THA [20]. But Noah et al. thought the TAL could not be routinely identified during surgery and when used for acetabular component orientation it was no more accurate than free-hand technique [23]. The remaining one compare TAL with mechanical angle guide device for acetabular component orientation. In conclusion, both TAL and mechanical angle guide device can effectively locate acetabular component. The TAL is patient-specific intraoperative landmark independent of patient position, while the mechanical angle guide device can lead to false assessments of acetabular component orientation [33].

Seven of the 19 (36.8%) studies included clinical benefit of using the TAL for intraoperative determination of the anteversion of acetabular component [9, 12, 21–[22, 28, 31]–32]. They consistently found that the TAL was a practical anatomical landmark for determining acetabular component orientation in THA. Kamal Deep et al. described the orientation of outer, middle, and inner margins of TAL with respect to anterior pelvic plane and Lewinnek’s safe zone. They showed that the inner margin of TAL provided the best opportunity to orient to acetabular component orientation [32].

### Rate of dislocations

Eleven of the 19 (57.9%) studies reported rate of dislocations by using the TAL as a landmark for the orientation of the acetabular component [12, 13, 17, 20–22, 28–30, 33]–34]. there was a total of 2,355 patients and 2,386 hips included, the average rate of dislocations was 0.75%, the follow-up time ranged from 1 to 96 months. There are three comparative studies involving dislocation rates [17, 20, 33]. In total, 90 patients and 90 hips were included in the TAL group. During follow-up, no postoperative dislocations occurred in all patients. A total of 93 patients and 94 hips were included in the control group, the average rate of dislocations was 2.13%.

## Discussion

In this systematic review we analyzed 19 articles to determine the significance of TAL as a landmark for the orientation of the acetabular component, and to identify proposed safe zone for acetabular component anteversion and inclination to reduce the risk of dislocation and impingement, achieve stability, maximize range of motion, improve survival. The results of the research indicate that the TAL can be used as a reference landmark to align the acetabular component in the safe zone for anteversion and inclination.

In THA, Lewinnek et al. reported that when the orientation of the acetabular component was set anteversion at 15º±10º and inclination at 40º±10º, the number of hip prosthesis dislocations evidently declined [6].Since then, the fixed angle was known as the ‘safe zone’ of Lewinnek et al. for which the surgeons had been pursuing as a reference standard. Taking Lewinnek’s safe zone as reference standard, 12 studies [9, 17, 21–26, 30, 32–34] identificated accuracy of TAL in the literature by imaging measurement methods, and agreed that using TAL as an intraoperative landmark was a simple and effective method for proper acetabular component orientation in THA (Table 2). K. Fujita et al. measured anteversion of TAL by aligning the inferomedial rim of acetabular component with the TAL using computer-assisted navigation during the operation, 94.6% (106 of 112 hips) were within the safe zone. They found that the TAL is a useful intraoperative landmark to implant the acetabular component within the safe zone. However, there are also individual differences that we should be aware of [25]. Kamal Deep et al. described the orientation of different parts of TAL for 99 patients relative to anterior pelvic plane. 17.17%, 28.28%,47.47% and 71.71% of acetabular component orientation were within Lewinnek’s safe zone, when acetabular component corresponds to acetabular rim, outer, middle, and inner margin of TAL respectively. They found that the inner margin of TAL provided the best opportunity to locate the acetabular component [32].

The TAL spans the inferior acetabular notch and extends to the lateral rim of the acetabulum, which is a useful intraoperative landmark to align the acetabular component in the safe zone for anteversion and inclination. But it is mostly used for normal or primary hip osteoarthritis [9, 35]. Some authors recognize that the TAL may not be a practical intraoperative landmark in the revision THA or in the presence of dysplastic hips [10]. Hirohito Abe et al. compared the anatomical anteversion of TAL by examing 80 hips with osteoarthritis secondary to hip dysplasia and 80 hips with osteonecrosis of the femoral head. There was a significant difference regarding the anatomical anteversion of TAL between groups [24]. Conversely, K. Fujita et al. compared 52 dysplastic hips with 60 non-dysplastic hips, and found that there was no significant difference regarding the anatomical anteversion of TAL between groups [25]. In the group-related comparison of acetabular component orientation guided by the TAL, there was no statistical difference in radiographic anteversion. However, there was a significant statistical difference in radiographic inclination between groups, and the pelves in the dysplastic hip group tended to tilt more anteriorly [22]. TAL is a good intraoperative landmark in hips with normal anatomy including osteonecrosis of the femoral head, TAL is not a reliable intraoperative landmark in hips with osteoarthritis secondary to dysplasia for determining optimal acetabular component orientation during THA [24]. Table 3 gives a summary of the results.

**Table 3:** Dysplastic group VS non-dysplastic group

	Dysplastic Group			Non-dysplastic group		
	Hideaki Miyoshi et al. (2012) [22]	Abe H et al. (2012) [24]	Fujita K et al. (2014) [25]	Hideaki Miyoshi et al. (2012) [22]	Abe H et al. (2012) [24]	Fujita K et al. (2014) [25]
Study Design	retrospective study	prospective study	prospective study	retrospective study	prospective study	prospective study
Patients N (hips)	15(15)	80(80)	(52)	14(15)	80(80)	(60)
Mean age (y)	63.5 (49–83)	54 (10, 34–81)	NA	69.7 (51–84)	47 (15,21–81)	NA
Sex (M/F)	(3/12)	(38/42)	NA	(4/10)	(38/42)	NA
Diagnosis	OA caused by dysplasia	OA caused by dysplasia	NA	OA,12 ON,1 RA, 2	ON	NA
Acetabular component Anteversion (P-Value)	21.5 ± 3.3 (15.7–27.3) 0.917	23(22–25) < 0.001	NA	21.4 ± 4.3 (14.5–28.8) NA	17(15–18) NA	NA
Acetabular component Inclination (P-Value)	42.1 ± 6.4 (30.9–54.1) 0.016	66(64–67) < 0.001	NA	35.1 ± 7.3 (22.1–50.7) NA	66(64–67) NA	NA
Pelvic tilt (°) (P-Value)	-3.9 ± 5.9 (-12.8-5.3) 0.074	5.9°±7.1 (–20–20) 0.8	NA	1.9 ± 9.0 (-13.7-14.2) NA	5.6°±6.4 (–12–19) NA	NA
TAL anteversion (°) (P-Value)	NA	16(14.1–17.8) < 0.001	21.1 ± 6.9 (7.7–39.3) 0.14	NA	5.7(3.8–7.5) NA	24.1 ± 9.4 (10.3–54.7) NA

M/F male/female, N number, TAL transverse acetabular ligament, OA osteoarthritis, ON osteonecrosis, RA rheumatoid arthritis, NA not applicable

The orientation of TAL varies greatly among individuals and is influenced by gender. The anteversion of TAL has a remarkable gender difference and is greater in females. 3 studies [24, 27, 32] reported the difference of TAL between gender (Table 4). Hirohito Abe et al. reported females in the group of hip osteoarthritis had considerably greater TAL anteversion than males, while there were no differences between the sexes in osteonecrosis of the femoral head group [24].

Andrew R. Griffin et al. report the mean anteversion angle of TAL for males and females was 19.0°±6.3°and 22.0°±7.4° respectively. Statistical analysis showed a significant difference between males and females for all acetabular and TAL anteversion angles [27].

Although the TAL was a practical anatomical landmark for determining acetabular component orientation in THA, some TALs were covered by soft tissue or osteophytes, so it may be difficult to visualise intraoperatively. Archbold *et* al divided TAL into 4 grades according to degree of the appearance [13]. 6 studies reported appearance of the TAL in the literature [12, 13, 17, 20, 22, 25], there were a total of 1306 hips included, of which 94.8% (N = 1238), the TAL can be visualized intraoperatively. To accurately expose the TAL in the THA, firstly, we can start with its anatomical structure. Osteotomy was performed on the femoral neck during THA, the femoral head is removed, two important anatomical structures are clearly visible: the ligamenta capitis femoris and the acetabular notch. The ligamenta capitis femoris is a flat triangular fibrous band in the capsule of the hip joint, with bases attached to the TAL and the sides of the acetabulum notch. The TAL spans the inferior acetabular notch and extends to the lateral rim of the acetabulum. Therefore, TAL can be found along the ligamenta capitis femoris and the notch of the acetabulum during surgery to improve TAL exposure. In addition, the acetabular lip loses cartilage at the acetabular notch, forming a TAL across the notch, so the TAL can also be exposed along the acetabular lip in the inferior part of the acetabulum. Secondly, acetabular osteophytes and soft tissues are two major barriers to TAL exposure. Special tools such as teardrop retractors, bone knives, and small acetabular reamers can be used to improve TAL exposure [34, 36]. Osteophytes should be removed gradually, and this needs to be done slowly and carefully to avoid TAL damage. If the TAL cannot be found, it may be inadvertently destroyed in the THA [14]. Finally, after a TAL is found during THA, an acetabular retractor or tear drop retractor can be placed beneath the TAL to avoid occlusion of the osteophytes and soft tissue surrounding the acetabular and fully expose the TAL [34]. Intraoperative spot and line markers can also be used to improve the TAL exposure [37].

**Table 4:** The difference of TAL between gender

Author(year)		Male	Female	P-Value
Abe H et al. (2012) [24]	Patients N	38	42	NA
	Anteversion (°)	11° (8.3–14)	20° (17–23)	< 0.001
A.R. Griffin (2012) [27]	Patients N	79	81	NA
	Anteversion (°)	19.0 ± 6.3	22.0 ± 7.4	0.001
Deep K et al. (2021) [32]	Patients N	41	58	NA
	Anteversion (°)	12.04 ± 8.07	16.89 ± 7.71	0.004

N number, NA not applicable

In addition, TAL anteversion is also influenced by acetabular anteversion. In the hips with retroverted or pauci-anteverted acetabulum, TAL should be cautiously used as an intraoperative landmark for acetabular component orientation. There was rarely a report to compare the anteversion angle between TAL and acetabulum by a direct measurement method. Byung-Ho Yoon et al. measured the anteversion of TAL in computed tomography arthrography and compared it with the anteversion of acetabulum, there was a significant correlation between two groups. In eight hips, the TAL anteversion (less than 5°) was outside of the safe zone, of which, the acetabular anteversion was also less than 5° in three hips [23].

Similarly, the TAL also has an individual variation influenced by the pelvic orientation, as well as disease and gender. In a few hips, TAL anteversion may be excessive, so we must pay attention to individual variations, especially in patients with severe pelvic tilt [24, 25]. Furthermore, pelvic malrotation can influence TAL-guided acetabular component orientation and result in different clinical outcomes after THA. Tingxian Ling et al. found that backward pelvis malrotation increased TAL-guided acetabular component inclination and anteversion, which contributed to outlier above the safe zone and increased the dislocation rates of the hips after THA by comparing normal pelvis group with backward pelvis malrotation group [30]. For the patients with abnormal pelvic orientation, acetabular component orientation should be performed individually instead of guiding by TAL.

TAL’s reliability still needs to be confirmed among landmarks of localization for the acetabular component in THA. In one study (8 cadaveric pelves,14 hips), anatomical anteversions of the TAL, labrum and horns were measured relative to the anterior pelvic plane by using a navigator sensor and an optoelectronic device. The study showed that TAL anteversion was outside the safe zone, the labrum anteversion was within the safe zone [7]. In the other study (160 patients,218 hips), anteversion angles of the four positions at acetabular rim were measured (superiorly to inferiorly) in relation to the anterior pelvic plane. The TAL anteversion angle was closest to the central rim section of the acetabulum which was exactly the main orientation of the acetabulum, while the superior rim section of the acetabulum was relatively retroverted and the inferior rim section of acetabulum was comparatively more anteverted [27].

No clinical studies using TAL as an intraoperative landmark for acetabular component orientation in patients with stiff spine were found. Most patients with ankylosing spondylitis are accompanied by bony ankylosis of the hip joint, and they have bony fusion of the hip joint. As a result, it is difficult to accurately determine the location of the acetabulum and the bony anatomical landmarks surrounding the acetabulum during surgery. At this point, it is also difficult to locate the location of the acetabular component with reference to the bony landmarks. In clinical work, we found that the bone fusion in the hip is mostly located in the upper and outer weight-bearing regions, while the soft tissue septum is located in the medial and lower acetabular notch, where the TAL is located. Therefore, for patients with hip ankylosis, using TAL as a landmark for the acetabular component in THA is a better option.

This systematic review was not without limitations. First, most studies included in the systematic review are limited by the low level of evidence and lack of long-term follow-up. Second, many of our studies were smaller case series of 21–100 hips and may have been under -powered to detect a statistically significant difference in adverse events. Therefore, more randomized controlled studies with large sample sizes are needed to allow us to make a valid comparison with a similar cohort to investigate the precision and accuracy of TAL as intraoperative landmark in THA. Although the above limitations exist, one of the strengths of this systematic review is that, for the first time, it provided a quantitative summary of the existing published literatures about TAL’s reliability as an intraoperative landmark for acetabular component orientation in THA. The results of our review indicate that TAL may play a more important role than previously as an intraoperative landmark for proper acetabular component orientation in THA.

## Conclusions

The TAL is a useful intraoperative landmark for the orientation of the acetabular component within the safe zone in THA, which can reliably be used to align the acetabular component in the safe zone for anteversion and inclination, and using TAL as an intraoperative landmark was a simple, effective method for proper acetabular component orientation in THA. However, the TAL has individual variation influenced by some risk factors, such as gender, abnormal pelvic orientation, dysplastic hips, retroverted or pauci-anteverted acetabulum. For the patients with these risk factors, TAL should be used cautiously as an intraoperative landmark for aligning acetabular component orientation during THA. In addition, more randomized controlled studies with larger numbers of patients are needed to make a valid comparison with a similar cohort to investigate the precision and accuracy of TAL as an intraoperative landmark in THA.

## Data Availability

All data generated or analysed during this study are included in this published article.
